# Acquired Brown Syndrome as a Postoperative Complication of Orbital Wall Fracture Repair with Metallic Mesh

**DOI:** 10.1177/22925503251371048

**Published:** 2025-08-29

**Authors:** Justin J. Lee, Nikhil Patil, Trent Schimmel, Matthew D. Benson, Joshua J. DeSerres

**Affiliations:** 1 Division of Plastic Surgery, Department of Surgery, 12357 University of Alberta, Edmonton, AB, Canada; 2 Department of Ophthalmology and Visual Sciences, 12357 University of Alberta, Edmonton, AB, Canada

**Keywords:** acquired brown syndrome, orbital fracture, superior oblique tendon-Sheath syndrome, Traumatisme craniofacial, Syndrome de Brown acquis, Fracture orbitaire, Syndrome de la gaine du tendon du muscle oblique supérieur

## Abstract

**Background:** Surgical repair of orbital fractures comes with risks. One rare risk is interference with the actions of the superior oblique tendon-muscle complex causing an acquired Brown syndrome. We present the case of a 45-year-old man who developed acquired Brown syndrome after undergoing repair of a large orbital floor and medial orbital wall fracture using a titanium mesh implant. A case report was prepared to discuss a rare surgical risk with open reduction internal fixation (ORIF) of an orbital wall fracture. **Methods:** A retrospective chart review was performed. **Results:** Post-operative ophthalmological assessment revealed persistent diplopia along with limitations of up-gaze particularly in the adducted position. Ultimately, the patient underwent surgical repositioning of the orbital implant, which seemingly released the superior oblique muscle-tendon complex, resolving most of the diplopia. No further treatment with prisms or strabismus surgery has been required. **Conclusions:** Acquired Brown syndrome is a potential risk of surgical repair of orbital fractures involving the medial orbital wall. Herein this case study, we describe a case of acquired Brown syndrome following ORIF of an orbital floor and medial wall fracture, which was alleviated with a revision surgery.

## Résumé

**Introduction :** La réparation chirurgicale des fractures orbitaires comporte des risques. Un risque rare est l’interférence avec les actions du complexe musculo-tendineux du muscle oblique supérieur entraînant un syndrome de Brown acquis. Nous présentons le cas d’un homme de 45 ans qui a développé un syndrome de Brown acquis après avoir subi une réparation d’une importante fracture du plancher orbitaire et de la paroi orbitaire médiale au moyen d’un implant en treillis de titane. Un rapport de cas a été préparé pour discuter d’un risque chirurgical rare de la fixation interne de réduction ouverte d’une fracture de la paroi orbitaire. **Méthodes :** Une revue rétrospective de dossier a été réalisée. **Résultats :** L’évaluation ophtalmologique postopératoire a révélé une diplopie persistante avec des limitations du regard vers le haut, notamment en adduction. Finalement, le patient a subi un repositionnement chirurgical de l’implant orbitaire qui semble avoir libéré le complexe musculo-tendineux du muscle oblique supérieur, faisant disparaître l’essentiel de la diplopie. Aucun traitement supplémentaire avec des prismes ou chirurgie du strabisme n’a été nécessaire. **Conclusions :** Le syndrome de Brown acquis est un risque possible de la fixation interne d’une fracture de la paroi orbitaire médiale. Dans cette étude de cas, nous décrivons la réflexion chirurgicale sur un syndrome de Brown acquis consécutif à une fixation interne de réduction ouverte de la paroi orbitaire médiale qui a été résolu grâce à une chirurgie de révision.

## Introduction

Orbital fracture reconstruction is a complex procedure requiring exposure of the fracture, release of herniated orbital tissue, and repair of the bony defect using an implant. Due to the intricate anatomy near the medial orbit, there's a risk of implant placement interfering with the superior oblique muscle (SO).

Restriction of this muscle-tendon unit is known as Brown syndrome, which can be congenital or acquired. Acquired cases may result from trauma, scarring, or surgical complications. While rare, some reports link Brown syndrome to glaucoma implant surgery,^1-3^ and even fewer associate it with titanium mesh used in orbital fracture repair.^4,5^

We present a case of a patient who developed acquired Brown syndrome after orbital wall reconstruction with a titanium mesh. This report aims to highlight the condition as a preventable complication with proper surgical technique.

## Case Report

A 45-year-old man presented to the emergency department (ED) with right-sided facial trauma. He had periorbital swelling, conjunctival injection, and enophthalmos. Slight restriction in upgaze of the right eye was noted. He had minimal vertical diplopia on upgaze, which was felt to be related to his periorbital edema. He had no history of ocular misalignment or strabismus surgery.

**Figure 1.**
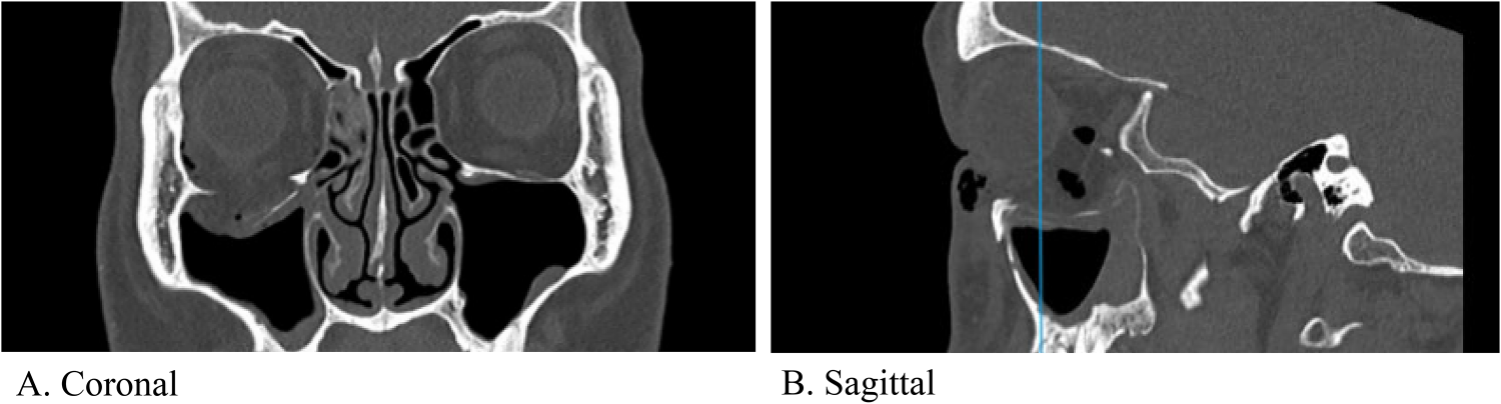
Pre-operative CT facial bones showing the orbital floor defect in coronal (A) and sagittal (B) views. Vertical line in the (B) panel reflects the corresponding slice in the coronal (A) view.

CT imaging showed a large orbital floor fracture (21 mm wide, 26.1 mm deep) and a medial wall fracture, with herniation of orbital contents into the maxillary sinus, inferior rectus rounding, and sinus hematoma (Figure 1). Due to the size of the defect, fracture location, and existing enophthalmos, the patient was scheduled for ORIF to prevent further enophthalmos and worsening diplopia.^6,7^

**Figure 2.**
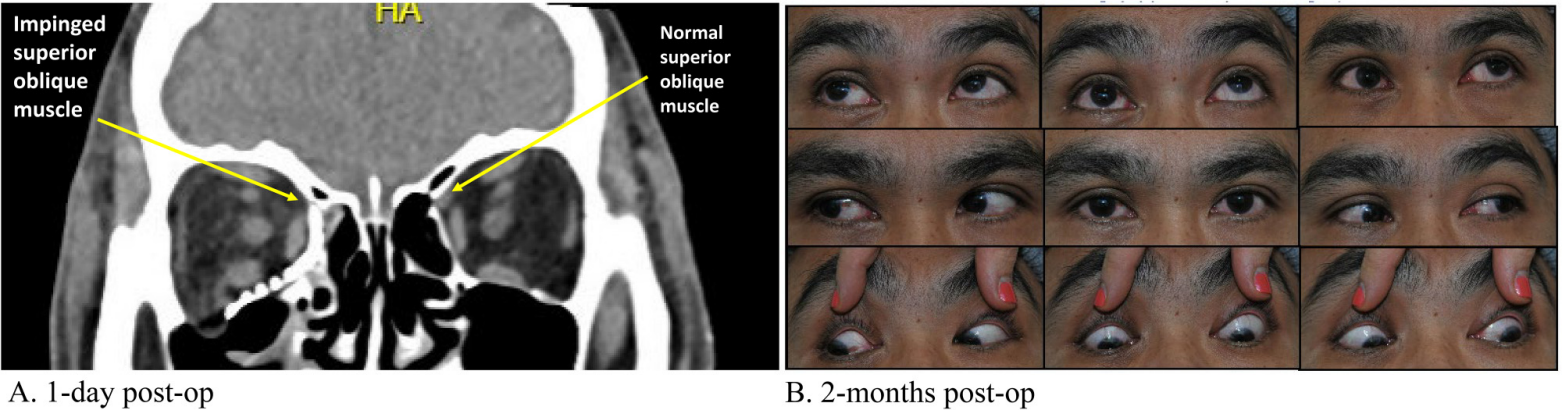
Post-operative CT facial bones (coronal view) (A) and orthoptic assessment (B) following initial ORIF. The metallic mesh appears to be impinging on the superior oblique muscle of the right eye (arrow) (A). Photos of the nine cardinal positions of gaze demonstrates limited upgaze of the right eye, particularly in adduction (B).

ORIF was performed four days following injury via transconjunctival preseptal approach with retro-caruncular extension and lateral canthotomy. A preformed DePuy Synthes orbital plate was placed and forced duction test was negative following plate positioning. On post-operative day (POD) one, the patient endorsed subjective diplopia in all directions of gaze with decreased ocular motility likely secondary to swelling. Routine post-operative CT facial bones showed satisfactory position of the implant with no concerning features (Figure 2A). He was discharged thereafter. On POD 18, the patient complained of ongoing vertical binocular diplopia with upgaze. He was noted to have a limitation in supraduction of the right eye. He was thereafter referred for ophthalmological assessment, where ocular motility examination confirmed a nearly complete inability to elevate the right eye in adduction, and a limitation in elevation in direct upgaze. Double Maddox rod testing confirmed 10 degrees of incyclotorsion. A reanalysis of the previously performed post-operative CT facial bones revealed a suspected restriction or impingement of the right SO as the likely etiology of the patient's symptoms (Figure 2A). Following collaborative discussion between plastic surgery and ophthalmology, a decision was made to conservatively monitor. With persistence of EOM restriction and diplopia at 2 months post-op, a diagnosis of acquired Brown syndrome was made (Figure 2B), and a decision was made to proceed with revision surgery.

**Figure 3.**
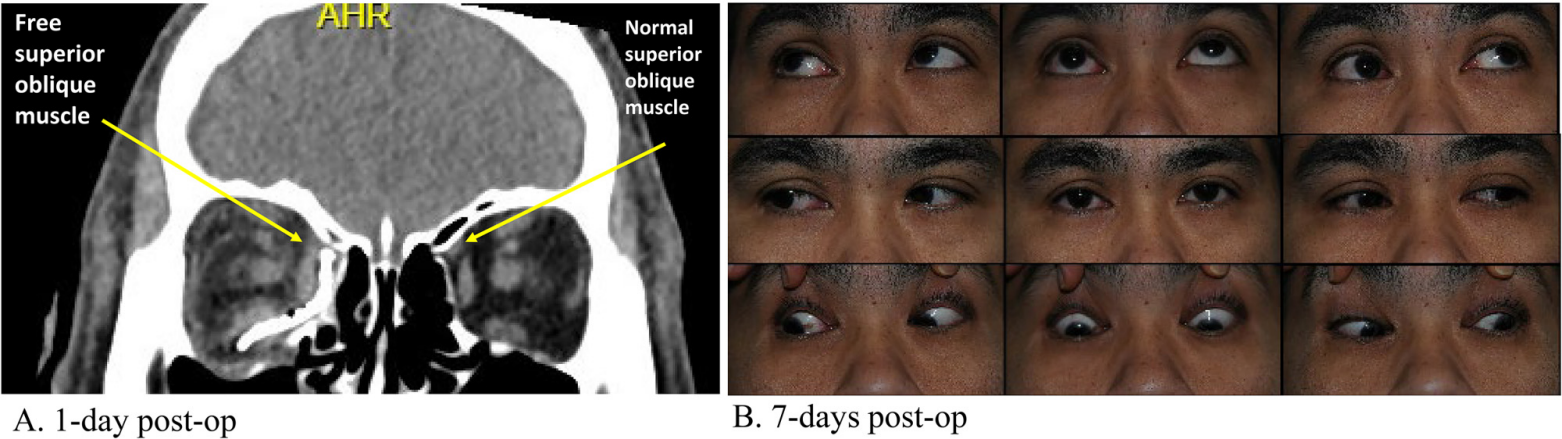
Post-operative CT facial bones (coronal view) (A) and orthoptic assessment (B) following hardware removal and revision of ORIF. The metallic mesh no longer appears to be impinging on the superior oblique muscle of the right eye (arrow) (A). Photos of the nine cardinal positions of gaze demonstrates improved alignment in primary position and an increased ability of the right eye to elevate in adduction (B).

Surgical revision was performed two months post-op via the same surgical approach and the implant was removed. Extensive soft tissue dissection was performed along the medial orbital wall and a new DuPuy Synthes preformed orbital plate was placed to reconstruct the defect. Intraoperative CT was used to ensure satisfactory positioning of the implant. An intra-op forced duction test revealed improved EOM, most notably with supraduction of the right eye when adducted. A repeat CT of the facial bones demonstrated improved plate position with no further imaging evidence of SO impingement (Figure 3A). At a follow-up on POD 7, the patient was still noted to have some restriction in elevation of the right eye in adduction (Figure 3B), however, his diplopia had significantly improved. At one-year post-op, the patient reported no diplopia in primary gaze, with diplopia only in eccentric gaze positions. He did not require any prisms or strabismus surgery, and no further medical interventions were required.

## Discussion

Herein, we describe a patient who underwent ORIF of a large two-wall orbital fracture secondary to trauma and subsequently developed an acquired Brown syndrome. This case report describes the post-operative course as well as the operative details.

The authors suspect that there was insufficient dissection of the periorbital soft tissue along the medial orbital wall, which led to inadequate implant positioning, resulting in impingement and restriction of the SO cascade. Key components of the revision surgery involved wide dissection, resulting in complete visualization of the medial orbital wall. Division of the contents of the anterior and posterior ethmoidal foramina should be performed to ensure adequate dissection and exposure for optimal implant placement along the medial wall. In addition, intra-operative imaging should be considered. Forced duction test should be performed after implant placement to assess all gaze directions, including incyclotorsion and excyclotorsion, to facilitate the identification of plate-related impingement that may require adjustment of the implant position.

This case highlights acquired Brown syndrome as a rare but significant complication of orbital wall ORIF. Surgeons should discuss this risk during consent and use meticulous dissection, careful implant placement, intra-op imaging, and thorough forced duction testing to minimize EOM impingement.

## References

[1] Ball SF Ellis GS Herrington RG Liang K . Brown's superior oblique tendon syndrome after Baerveldt glaucoma implant. Arch Ophthalmol. 1992;110(10):1368. doi:10.1001/archopht.1992.01080220030010 1417532

[2] Coats DK Paysse EA Orenga-Nania S . Acquired Pseudo-Brown's syndrome immediately following Ahmed valve glaucoma implant. Ophthalmic Surg Lasers. 1999;30(5):396‐397. 10334029

[3] Dobler AA Sondhi N Cantor LB Ku S . Acquired Brown's syndrome after a double-plate Molteno implant. Am J Ophthalmol. 1993;116(5):641‐642. doi:10.1016/s0002-9394(14)73209-x 8238227

[4] Ji SY Yoo JH Ha W Lee JW Yang WS . Three cases of acquired simulated Brown syndrome after Blowout fracture operations. Arch Plast Surg. 2015;42(03):346‐350. doi:10.5999/aps.2015.42.3.346 26015892 PMC4439596

[5] Hwang JU Lim HT . Acquired simulated Brown syndrome following surgical repair of medial orbital wall fracture. Korean J Ophthalmol. 2005;19(1):80‐83. doi:10.3341/kjo.2005.19.1.80 15929493

[6] De Ruiter BJ Kotha VS Lalezar FD , et al. Orbital Index: A novel comprehensive quantitative tool for prediction of delayed enophthalmos in orbital floor fracture management. Plast Reconstr Surg. 2022;150(3):625e‐629e. 10.1097/PRS.0000000000009428 35791257

[7] DeSerres JJ Budning A Antonyshyn OM . Current management of late posttraumatic enophthalmos. Plast Reconstr Surg. 2022;150(4):888e‐902e. 36170440 10.1097/PRS.0000000000009471

